# New *V. cholerae *atypical El Tor variant emerged during the 2006 epidemic outbreak in Angola

**DOI:** 10.1186/1471-2180-11-130

**Published:** 2011-06-13

**Authors:** Daniela Ceccarelli, Matteo Spagnoletti, Donatella Bacciu, Piero Cappuccinelli, Mauro M Colombo

**Affiliations:** 1 Dipartimento di Biologia e Biotecnologie Charles Darwin, Sapienza Università di Roma, Rome, 00185, Italy; 2 Dipartimento di Scienze Biomediche, Università degli Studi di Sassari, Sassari, 07100, Italy

## Abstract

**Background:**

*V. cholerae *is the etiological agent of cholera, a major public health concern in most developing countries. Virulence of *V. cholerae *relies on the powerful cholera toxin, encoded by the CTX prophage. The emergence of new pathogenic variants in the recent years has been mostly associated with new CTX prophage rearrangements.

**Results:**

In this retrospective study, we show that the epidemic *V. cholerae *O1 El Tor strain responsible for the 2006 outbreak in Angola is clonally and genetically different from El Tor strains circulating in the 1990s in the same area. Strains from 2006 carry ICE*Vch*Ang3 of the SXT/R391 family. This ICE is associated with a narrower multidrug resistance profile compared to the one conferred by plasmid p3iANG to strains of the 1990s. The CTX prophage carried by 2006 El Tor strains is characterized by *rstR^ET ^*and *ctxB^Cla ^*alleles organized in a RS1-RS2-Core array on chromosome I. Interestingly, the newly emerging atypical strain belongs to a clade previously known to comprise only clinical isolates from the Indian subcontinent that also contain the same ICE of the SXT/R391 family.

**Conclusions:**

Our findings remark the appearance of a novel *V. cholerae *epidemic variant in Africa with a new CTXΦ arrangement previously described only in the Indian Subcontinent.

## Background

*Vibrio cholerae *is the etiological agent of the severe watery diarrhoeal disease known as cholera, a major public health concern in most developing countries.

More than 200 serogroups have been described on the basis of different somatic O antigens [1], but only serogroups O1 and O139 have the ability to cause harsh epidemics. Serogroup O1 is further divided into two main biotypes, Classical and the 7^th ^pandemic El Tor. Beside their phenotypic characteristics, differences in specific genetic markers, such as toxin structure, confer distinct features to these biotypes.

Pathogenic *V. cholerae *strains carry the genes encoding the cholera toxin (CT) on the CTXΦ prophage. Different CTXΦ arrangements have been described within the O1 serogroup [2]. These arrangements depend on the genotype of the CT gene *ctxB *and on the organization and chromosomal location of several gene clusters of phage origin, namely the core, RS2, and RS1 [2]. Although the Classical biotype is considered extinct, new El Tor strains holding the Classical *ctxB *allele, generically labeled as atypical El Tor (including hybrid El Tor, altered El Tor and Mozambique variants) [2], were identified from 1993 to date mostly in Asia [3-8] with few cases in Africa [5,9,10]. The atypical variants are characterized by a new CTXΦ arrangement, holding El Tor and/or Classical alleles of *rstR *and *ctxB *genes [2]. As a consequence of these genetic arrangements in CTX prophage, toxigenic *V. cholerae *O1 El Tor strains have changed in the last 20 years. Initially, atypical variants were only sporadically identified in the Indian Subcontinent along with prototype El Tor. However they are now in the process of replacing it worldwide [2].

Prototype El Tor strains often contain multi-resistant conjugative plasmids [11] whereas O139 and atypical O1 El Tor *V. cholerae *epidemic strains usually harbor Integrative Conjugative Elements (ICEs) of the SXT/R391 family [12].

SXT/R391 ICEs are self-transmissible mobile elements, ranging in size from 79 to 108 kb, able to integrate into the host bacterial chromosome and to transfer by conjugation. They are recognized for their important role in bacterial genome plasticity [13] and as vectors of antibiotic resistance and alternative metabolic pathways [12]. The name of the SXT/R391 family originates from elements SXT^MO10 ^and R391, respectively discovered in clinical strains of *Vibrio cholerae *in India [14] and *Providencia rettgeri *in South Africa [15]. The two elements are associated with different multi-resistance profiles: chloramphenicol, streptomycin, sulfamethoxazole, and trimethoprim for SXT^MO10^, and kanamycin, and mercury for R391 [12]. They share a highly conserved genetic backbone encoding their integration/excision, conjugative transfer, and regulation, but also contain variable DNA found in five insertion sites of the backbone [12]. Each ICE of the family holds specific genes scattered in the conserved sequence that code for resistance to antibiotics and heavy metals, new toxin/antitoxin systems, restriction/modification systems, and alternative metabolic pathways [12]. To date more than 50 ICEs have been identified and grouped within the SXT/R391 family, most of them discovered in *V. cholerae *strains.

To date, only a few SXT-related ICEs were identified in Africa, most of them through the characterization of the integrase *int_SXT_*. Only ICE*Vch*Moz10 from Mozambique (2004) has been completely sequenced and annotated [12]. This ICE has no close relative in Africa except its sibling ICE*Vch*Ban9 isolated in Bangladesh (1994), suggesting the possible spread of SXT-related ICEs between the two continents in recent times. Although the use of horizontally-transferred elements as genetic markers for strain discrimination might appear risky, we recently showed the existence of an ICE/strain association in epidemic *V. cholerae *strains circulating in the Indian Subcontinent [16]. The association between ICE and *V. cholerae *reflects the classification proposed by Chun and colleagues to describe homologous intraspecific groups of *V. cholerae *based on the whole genome alignment of 23 strains isolated over the past 100 years [17].

In this retrospective study, we analysed *V. cholerae *O1 clinical strains isolated in Luanda (Angola) in 2006. Angola is an endemic area for cholera and was subjected to two major epidemic events in the past three decades. The first outbreak (1987-1993) [18] was followed by a thirteen year remission phase until cholera reemerged in 2006 in one of the most severe epidemic outbreaks of the last decade, counting about 240.000 cases [19].

Here we demonstrate that the *V. cholerae *O1 El Tor strain responsible for the 2006 Angolan outbreak is an atypical O1 El Tor variant previously detected only in Asia [3]. This variant is significantly different from those isolated during previous cholera outbreaks in the 1990s in the same geographic area. Indeed, it holds a peculiar CTXΦ array and the SXT-like element ICE*Vch*Ang3. Ribotype analysis suggests that this strain might have spread to West Africa from the Indian Subcontinent.

## Methods

### Bacterial strains, susceptibility tests and transfer of drug resistances

We analyzed *V. cholerae *strains isolated in Angola or India between 1992 and 2006 (Table 1). All strains were isolated from stool samples and/or rectal swabs from patients, and after isolation on thiosulfate citrate bile sucrose agar and biochemical identification, bacterial strains were routinely grown in Luria-Bertani (LB) or agar plates at 37°C and maintained at -80°in LB broth containing 30% (vol/vol) glycerol.

**Table 1:** *V. cholerae *O1 strains analyzed in this study

	Isolation							
							
Strain	Place	Year	Antibiotic resistance profile	Antibiotic resistance genes	ICE content	CTXΦ array	Ribotype	Reference
VC175	Angola (Luanda)	2006	Ap, Pn, Sm, Su, Tp	*floR, strA, strB, dfrA1, sulII^b^*	ICE*Vch*Ang3	B	R1	This study
VC189	Angola (Luanda)	2006	Ap, Pn, Sm, Su, Tp	*floR, strA, strB, dfrA1, sulII^b^*	ICE*Vch*Ang3	B	R1	This study
VC582	Angola (Luanda)	1992	Ap, Cm, Kn, Pn, Sm, Sp, Su, Tc, Tp^a^	*aph, tetG, cat1, blaP1, dfrA15, aadA8, sul2^c^*	-	A	R2	[11]
VC1383	Angola (Benguela)	1994	Ap, Cm, Kn, Pn, Sm, Sp, Su, Tc, Tp^a^	*aph, tetG, cat1, blaP1, dfrA15, aadA8, sul2^c^*	-	A	R3	[11]
VC547	Angola (Bengo river)	1994	Ap, Cm, Kn, Pn, Sm, Sp, Su, Tc, Tp^a^	*aph, tetG, cat1, blaP1, dfrA15, aadA8, sul2*^c^	-	A	R4	[11]
VC7452	India (Sevagram)	1995	Ap, Nx, Pn, Sm, Sp, Su, Tp	*floR, strA, strB, dfrA1, sulII^b^*	ICE*Vch*Ind5^d^	B	R1	[16]
VC15699	India (Sevagram)	1999	Ap, Nx, Pn, Sm, Sp, Su, Tp	*floR, strA, strB, dfrA1, sulII^b^*	ICE*Vch*Ind5	B	R1	[16]
VC9258	India (Sevagram)	1999	Ap, Nx, Pn, Sm, Sp, Su, Tp	*floR, strA, strB, dfrA1, sulII^b^*	ICE*Vch*Ind5	B	R1	[16]

^a^Resistance profile conferred by conjugative plasmid p3iANG [11]; ^b^located on the ICE; ^c^located on p3iANG; ^d^ICE fully sequenced. Abbreviations: Ap, ampicillin; Cm, chloramphenicol; Kn, kanamycin; Nx, nalidixic acid; Pn, penicillin; Sm, streptomycin; Sp, spectinomycin; Su, sulfamethoxazole; Tc, tetracycline; Tp, trimethoprim.

Antimicrobial susceptibility was tested at the following concentrations: ampicillin (Ap), 100 μg/ml; chloramphenicol (Cm), 20 μg/ml; kanamycin (Km), 50 μg/ml; nalidixic acid (Nx), 40 μg/ml; penicillin (Pn), 20 μg/ml; rifampin (Rf), 100 μg/ml; spectinomycin (Sp), 50 μg/ml; streptomycin (Sm), 50 μg/ml; sulfamethoxazole (Su), 160 μg/ml; tetracycline (Tc), 12 μg/ml; and trimethoprim (Tm), 32 μg/ml. Antibiotic concentrations were defined according to their MIC breakpoints as previously described [18,20] and were included in ISO sensitest (Oxoid) agar plates. Bacterial strains were spotted onto the plates as previously described [11].

Conjugation assays were used to transfer ICE*Vch*Ang3 from *V. cholerae *into rifampin-resistant derivatives of *E. coli *803 strain. Mating assays were performed by mixing equal volumes of overnight cultures of donor and recipient strains. Briefly, the cells were harvested by centrifugation and resuspended in a 1/20 volume of LB broth. Cell suspensions were poured onto LB agar plates and incubated at 37°C for 6 h. The cells were then resuspended in 1 ml of LB medium, and serial dilutions were plated onto appropriate selective media to determine the numbers of donors, recipients, and exconjugants. Frequency of transfer was expressed as the number of exconjugant cells per donor cells in the mating mixture at the time of plating.

*V. cholerae *O139 MO10 [14], *V. cholerae *E4:ICE*Vch*Ind1 [21], *V. cholerae *O1 VC20 [22], *V. cholerae *N16961 [23], *V. cholerae *O1 CO840 [22], *V. cholerae *O1 VC7452, VC15699, and VC9258 isolated in India (Maharashtra) [16], and *E. coli *AB1157:R391 [24] were appropriately used as negative or positive controls for class 1 integrons, ICE, *tcpA*, and *rstR *detection, CTXΦ array and ribotype analysis.

### Molecular biology procedures

Bacterial DNA for PCR analysis was prepared with a Wizard Genomic DNA Purification kit (Promega). Amplicons to be sequenced were directly purified from PCR or extracted from agarose gel by Wizard SV Gel and PCR Clean-up System (Promega) according to the manufacturer's instructions. DNA sequences were determined by BMR Genomics (Padova, Italy).

Class 1 integron detection was performed by PCR amplification with specific primer pairs as previously described [11]. ICEs of the SXT/R391 family were screened by PCR analysis, using 17 specific primer pairs previously described by our group [25,26]. *int*_SXT_, *prfC*/SXT^MO10 ^right junction, *floR, strA, strB, sul2, dfrA18, dfrA1, rumAB *operon, *traI, traC, setR*, and Hotspots or Variable Regions *s026/traI, s043/traL, traA/s054, s073/traF *and *traG/eex *were screened. A second set of 15 primer pairs designed on the specific sequences of ICE*Vch*Ind5 [16] were used to detect ICE*Vch*Ind5 and ICE*Vch*Ang3 specific Hotspots and Variable Regions.

All PCR reactions were set in a 50-μl volume of reaction buffer containing 1 U of *Taq *polymerase as directed by the manufacturer (Promega).

### Ribotype analysis

Ribotyping of *V. cholerae *strains was performed by *Bgl*I restriction of chromosomal DNA with fluorescent-labeled 16S and 23S DNA (Gene Images 3540 RPn3510, Amersham) generated by reverse transcriptase polymerase chain reaction of ribosomal RNAs, as already described [25].

### CTX array analysis and *ctxB, tcpA, rstR *biotype characterization

**Figure 1:**
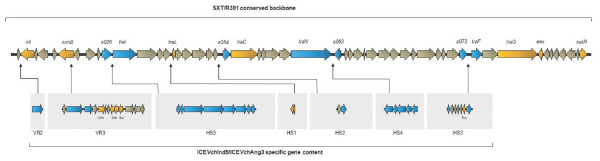
**ICE*Vch*Ang3 genetic structure**. Schematic linear representation (adapted from Wozniak et al., 2009) of the genes amplified by PCR to define the molecular structure of ICE*Vch*Ang3. The upper line represents the conserved backbone of the SXT/R391 family members. The black arrows indicate insertion sites for ICE*Vch*Ind5/ICE*Vch*Ang3 specific gene content. Genes in orange were tested with primer set A. Genes in blue were tested with primer set B. Genes not tested are shown in grey. VR: Variable Region; HS: Hotspot. GenBank accession no. of the full sequence of ICE*Vch*Ind5 is GQ463142[12].

The structure of CTX array was determined by multiple PCR analysis (Table 2) and by Southern Blot hybridization. The genetic structure of the two CTX prophage arrays described in Figure 1 was determined using the primers described in Table 2. Briefly, combination of primers tlcF/rstAR, tlcF/rstCR, rstCF/rstAR, ctxAF/rstAR, rstCF/rtxR and ctxAF/rtxR were used to detect the presence of CTXΦ on chromosome 1 and to determine the position of the RS1 element (see Additional file 1 Table S1 for complete amplicon profiles). The absence of CTXΦ or RS1 on chromosome 2 was established using primers chr2F/chr2R. Primers ctxAF/cepR were used to determine the presence of CTX tandem arrays.

**Table 2:** Primers used to determine CTX prophage array structure

Primer	Nucleotide sequence (5' to 3')	**Position (GenBank Accession no. **NC_002505-6)
tlcF	CCAAAACAACAGAAGCAACAGAGCAACG	1574460-1574487
rstCF	GGCGCTTATACAGACGAAATCGCTC	1564180-1564201
rstCR	AGCGCCTGAACGCAGATATAAA	1564290-1564311
rstAR	CGACAAAAACAAACGGAGAAGCGT	1572748-1572771
ctxAF	CTCAGACGGGATTTGTTAGGCACG	1567895-1567918
rtxR	CAAGCTGCGATCAGCATGGCGTGGTC	1563652-1563671
cepR	CAGTGTTTTGGTGACTTCCGT	1571101-1571121
chr2F	CTCACGCTGAACAGCAAGTC	507564-507583
chr2R	AAACCGGGAGAAGTGATTGC	509487-509506

Three previously described primer sets were used to detect: (i) Classical, El Tor, or Kolkata type *rstR *gene [27], (ii) *ctxB *genotype sequencing [28], (iii) and Classical or El Tor biotypes for *tcpA *[29].

PCR results on organization and location of CTXΦ on chromosome 1 were further confirmed by Southern Blot hybridization assays. DNA probes were produced by PCR using the chromosomal DNA of *V. cholerae *strain N16961 as template: *ctxA *gene (564 bp) with primers CTX-2 (CGGGCAGATTCTAGACCTCCTG) and CTX-3 (CGATGATCTTGGAGCATTCCCAC); *rstA *gene (789 bp) with primers rstA1F (AAACCTGCAAAATACCCCT) and rstA1R (ACAACTCGATACAAACGCT). Probes for hybridization were labeled with alkaline phosphatase with AlkPhos Direct™ Labelling and Detection System with CDP-*Star™ *kit (Ge Healthcare), according to manufacturer's instructions.

Strains were cultured in Luria-Bertani medium and 1 ml of culture was used to extract and purify the genomic DNA using the DNeasy Blood & Tissue Kit (Qiagen). Aliquots of the extracted DNA (1,5 μg) were digested with *EcoR*V for CTXΦ element restriction fragment length polymorphism analysis. The digested fragments were separated by agarose gel electrophoresis (1% gel) and were blotted on nitrocellulose membranes using standard methods [30]. Southern blots were hybridized O/N with *ctxA *or *rstA *labeled probes, and washed under stringent conditions, according to manufacturer's instructions. Addition of CDP-Star Detection Reagent was followed by 10 min incubation, and autoradiography (20 min to 1 h) was performed to generate a signal.

### Nucleotide sequence accession numbers

Nucleotide sequences of *ctxB *genes were deposited in GenBank under accession no. HQ599507 (*V. cholerae *1383), HQ599508 (*V. cholerae *7452), HQ599509 (*V. cholerae *547), HQ599510 (*V. cholerae *582), and HQ599511 (*V. cholerae *175).

## Results

### *V. cholerae *strains from 2006 show reduced resistance profile compared to previous epidemic strains

We analyzed two *V. cholerae *O1 El Tor clinical strains, VC175 and VC189 (Table 1), isolated at the Luanda Central Hospital (Angola). These strains were collected during the peak (May) of the cholera outbreak reported in Angola in 2006.

The two strains were sensitive to tetracycline, chloramphenicol, and kanamycin but showed a multiresistant profile to ampicillin, penicillin, streptomycin, trimethoprim, and sulfamethoxazole (see Table 1 for complete phenotype and genotype). Despite this significant multidrug resistance, these strains showed a narrower resistance profile compared to those isolated in the previous 1987-1993 cholera epidemic, which were also resistant to tetracycline, chloramphenicol, spectinomycin and kanamycin [11]. We found no evidence for the presence of conjugative plasmids or class 1 integrons in the 2006 strains analyzed (data not shown), which might explain their reduced drug resistance profile. Indeed, strains from 1987-1993 were associated with the conjugative plasmid p3iANG that holds genes encoding the resistance to tetracycline, chloramphenicol, kanamycin, and spectinomycin [11].

### ICE*Vch*Ang3 is a sibling of ICE*Vch*Ind5

We assessed the presence of SXT/R391 family ICEs since they are a major cause of antibiotic resistance spread among *V. cholerae *strains. Both strains were *int*_SXT_^+^, were shown to contain an ICE integrated into the *prfC *gene, and contained the conserved genes *traI, traC *and *setR*, respectively encoding a putative relaxase, a putative conjugation coupling protein, and a transcriptional repressor found in all SXT/R391 family members [31]. Based on these results we included this ICE in the SXT/R391 family and named it ICE*Vch*Ang3 according to the accepted nomenclature [32].

SXT/R391 ICEs exhibit significant genetic polymorphisms in hotspot content [12]. We used a first set of primers (primer set A), designed to discriminate between SXT^MO10 ^and R391 specific sequences [25], in order to prove the identity of the ICE circulating in the 2006 Angolan strains. Genes *floR, strA, strB, sul2, dfrA18, dfrA1*, the *rumAB *operon, and Hotspots or Variable Regions *s026/traI, s043/traL, traA/s054, s073/traF *and *traG/eex *were screened.

The 2006 strains exhibited the same SXT^MO10^/R391 hybrid ICE pattern. Intergenic regions *traG*/*eex *(Variable Region 4) and *traA/s054 *(Hotspot 2) showed the molecular arrangement described in SXT^MO10^, whereas region *s043/traL *(Hotspot 1) was organized as in R391. Variable Region 3, inserted into the *rumB *locus, contained genes that mediate resistance to chloramphenicol, streptomycin and sulfamethoxazole: *floR, strA, strB, sul2*. Interestingly, ICE*Vch*Ang3 lacks *dfr18*, the gene conferring resistance to trimethoprim found in SXT^MO10^, and carries instead *dfrA1 *in Hotspot 3. This preliminary analysis revealed that ICE*Vch*Ang3 exhibits a hybrid genetic content similar to that of the completely sequenced ICE*Vch*Ind5, the most widespread ICE circulating in *V. cholerae *El Tor O1 strains in the Indian Subcontinent [16].

Given these similarities we analyzed ICE*Vch*Ang3 using a second set of primers (primer set B) previously designed to assess the hotspot content of ICE*Vch*Ind5 [16]. This analysis confirmed that all the peculiar insertions found in ICE*Vch*Ind5 were also present in ICE*Vch*Ang3: (i) a gene encoding a protein similar to the *E. coli *dam-directed mismatch repair protein MutL (Variable Region 2); (ii) intI9 integron (Hotspot 3); (iii) a possible transposon of the IS21 family (Hotspot 4); and (iv) a 14.8-kb hypothetical operon of unknown function (Hotspot 5). On account of our results and of the common backbone shared by SXT/R391 ICEs (~65% of the ICE), we are confident that ICE*Vch*Ang3 is a sibling of ICE*Vch*Ind5 [16]. A map (not to scale) of ICE*Vch*Ang3 is shown in Figure 1.

We performed mating experiments to assess the ability of ICE*Vch*Ang3 to transfer by conjugation between *V. cholerae *strain VC 175 or VC 189 and *E. coli *803Rif. The frequency of transfer of ICE*Vch*Ang3 was 4,4 X 10^-5^, a frequency of transfer similar to that of most of the ICEs of this family. Ten *E. coli *exconjugant colonies were tested and proved to be positive for the presence of *int_SXT_*, confirming the mobilization of ICE*Vch*Ang3.

### A new CTXΦ array in Africa

The variability of CTXΦ and the emergence of atypical El Tor variants in the ongoing 7^th ^pandemic [2] les us to analyze the organization of CTXΦ arrays and the presence of different alleles of *ctxB, rstR *and *tcpA *genes. The genetic structure of CTX prophage in the genome of the Angolan isolates from both epidemic events was determined by multiple PCR analysis, hybridization, and sequencing, when required.

**Figure 2:**
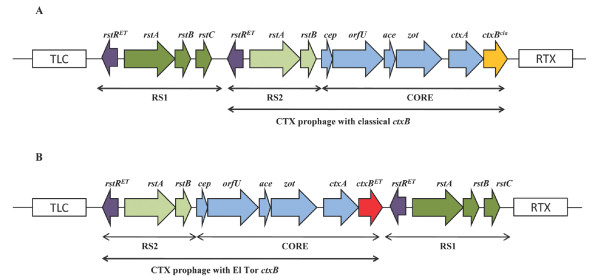
**Comparison of the genetic structures of the two CTX prophage arrays identified in the *V. cholerae *strains under study**. Both prophages are integrated into the large chromosome. Arrows indicate the transcription direction of each gene. (A) CTX prophage array profile A: RS1-RS2-CORE; (B) CTX prophage array profile B: RS2-CORE-RS1. Map is not to scale. *rstR^ET ^*(purple arrow): El Tor type *rtsR; ctxB^ET ^*(red arrow): El Tor type *ctxB; ctxB^cla ^*(yellow arrow): Classical type *ctxB*; TLC: toxin-linked cryptic plasmid; RTX: RTX (repeat in toxin) gene cluster.

Combining the results obtained by multiple PCR analysis and hybridization we were able to show that the strains analyzed contained two distinct CTXΦ arrays (A and B), both of which were found integrated in the large chromosome (Figure 2, Additional file 1 Table S1). These strains also proved to be negative for any CTXΦ integration on the small chromosome and devoid of CTX tandem arrays as detected by primer pairs chr2F/chr2R and ctxAF/cepR, respectively. The Angolan strains isolated in 2006 (VC 175 and VC 189) belonged to profile A, in which the RS1 element is followed by CTXΦ, both being located between the toxin-linked cryptic (TLC) element and the chromosomal RTX (repeat in toxin) gene cluster (Figure 2a). In contrast, strains from the first outbreak (1987-1993) contained CTXΦ followed by the RS1 element (profile B) (Figure 2b). Both CTXΦ arrays were characterized by El Tor type *rstR *genes (both in RS1 and RS2) but showed a noteworthy difference in their *ctxB *genotype (Table 3). CTXΦ arrays belonging to profile A contained a histidine and a threonine at the 39^th ^and 68^th ^amino acid positions, respectively, which are representative of Classical genotype 1 CtxB. The CTXΦ arrays belonging to profile B held a tyrosine, a phenylalanine and an isoleucine at positions 39^th^, 46^th ^and 68^th^, respectively, typical of an El Tor genotype 3 CtxB.

**Table 3:** Biotype characterization and *ctxB *genotype comparison of *V. cholerae *O1 isolates from Angola and India

Strain	*rstR*	*tcpA*	*ctxB*	
			
			Genotype^a^	Amino acid position^b^
VC582	ET	ET	3 (ET)	20 (His); 24 (Gln); 28 (Asp); 34 (His); 39 (Tyr); 46 (Phe); 55 (Lys); 68 (Ile)
VC547	ET	ET	3 (ET)	20 (His); 24 (Gln); 28 (Asp); 34 (His); 39 (Tyr); 46 (Phe); 55 (Lys); 68 (Ile)
VC1383	ET	ET	3 (ET)	20 (His); 24 (Gln); 28 (Asp); 34 (His); 39 (Tyr); 46 (Phe); 55 (Lys); 68 (Ile)
VC175	ET	ET	1 (Cla)	20 (His); 24 (Gln); 28 (Asp); 34 (His); 39 (His); 46 (Phe); 55 (Lys); 68 (Thr)
VC7452	ET	ET	1 (Cla)	20 (His); 24 (Gln); 28 (Asp); 34 (His); 39 (His); 46 (Phe); 55 (Lys); 68 (Thr)

Cla, Classical type; ET, El Tor type; ^a^According to *ctxB *genotyping by Safa et al., 2010 [2]; ^b^Nucleotide position +1 corresponds to the A of the ATG start codon in *ctxB*.

### Angolan and Indian strains share the same clonal origin

In order to verify their clonal relationship, we analysed by ribotyping the strains from the two Angolan epidemics of the 1990s and of 2006, as well as the Indian strains collected from 1993 to 2005 (Table 1) [16]. Strains from 1987-1993 outbreak (VC582, VC1383 and VC547) were chosen according to their epidemiological role (clinical or environmental isolate) and the presence of plasmid p3iANG [11].

Angolan strains isolated between 1992 and 1994 showed an assorted ribotype profile: clinical strains VC582 and VC1383 were characterized by profiles R2 (2.3,4.2, 4.6, 5.7, 6.0 kb) and R3 (2.3,4.2, 4.6, 5.7, 6.0, 9.6, 18.0 kb), respectively, and environmental isolate VC547 by a third completely different profile R4 (1.0, 1.4, 1.6, 1.8, 2.0, 2.2, 2.4, 3.8, 5.5 kb). This heterogeneity is not surprising if we consider the Angolan clinical strains on a larger sample scale. Indeed, our data showed that there was a clonal shift in Angola from 1992 to 1993/1994 with consequent change of ribotype (D.C personal communication) that can explain the discrepancies observed here. Strains VC175 and VC189 isolated in 2006 were characterized by the same ribotype profile R1 (2.3, 4.2, 5.8, 6.1, 6.3, 8.5, 9.4, 10.8, 22.0 kb) which corresponds to the ribotype profile of the Indian strains carrying ICE*Vch*Ind5 [16], suggesting a common clonal origin.

## Discussion

2006 was a crucial year for cholera worldwide. The number of reported cases was higher than ever and exceeded the levels of the late 1990s. Major outbreaks affected some of the largest African countries, including Angola, which reported to WHO one of the most exceptional epidemics experienced in Africa in the last decade [19].

This is the first study on the causative agent of this dramatic outbreak and our analysis revealed significant differences between the Angolan strains of 2006 and those isolated in the previous 1987-1993 cholera epidemic. The 1987-1993 epidemic was the longest in Angolan history and the *V. cholerae *epidemic strains were characterized by the presence of the conjugative plasmid p3iANG that carries three class 1 integrons [11]. Interestingly, the strains from the 2006 outbreak lack p3iANG but harbor an SXT-like ICE sibling of ICE*Vch*Ind5, previously described only in Asian *V. cholerae *strains [16]. The gene content of ICE*Vch*Ang3 comprises elements shared with SXT^MO10^, R391, ICE*Vch*Ban9, and ICE*Pda*Spa1, alongside some unique insertions of unknown function that might provide the strain with increased fitness. In light of its genetic content we included ICE*Vch*Ang3 in the subgroup of SXT/R391 ICEs that characterizes *V. cholerae *O1 El Tor strains circulating in several epidemic areas of the Indian Subcontinent, of which ICE*Vch*Ind5 is the reference ICE [12,16].

Beside the analysis of the Mozambican variant, extensive studies of CTXΦ arrangements in *V. cholerae *strains isolated in Africa lack so far.

Our analysis reports that the strains of the 2006 outbreak contain an RS1-CTX array on the large chromosome with a classical *ctxB *allele, which classifies them as *V. cholerae *O1 altered El Tor. This variant was responsible for major epidemics in India in 2004-2006 [3] and in Vietnam in 2007 [8]. It is considered as prevalent in Asia nowadays [33,34] and forms a monophyletic group with other variants of the 7^th ^pandemic clade [17]. This variant arose in the Indian Subcontinent at the beginning of the 90s and slowly diffused to Asian countries [6,7]. The possible spread to Africa was only suggested [3,33] and some authors gave partial evidences supporting this hypothesis by strain ribotyping [22] or *ctxB *genotyping [5]. With this work we ascertain the presence of this atypical El Tor variant in Africa and demonstrate it holds the responsibility for the 2006 cholera epidemic in Angola.

The Angolan variant is the second example of atypical El Tor variant described in Austral Africa, the first being the Mozambican strain B33 [9]. However, this variant is different from the Angolan one, since it holds a tandem CTXΦ array on the small chromosome [33], contains a different ICE (ICE*Vch*Moz10) [12], and is closely related to the Bangladeshi strain MJ-1236 [7,17].

Unlike B33 whose progenitor was identified as a Kolkata hybrid strain from 1992 [35], we have no clear information on how the variant we found in Angola penetrated Austral Africa. We can speculate that it arrived from the Indian Subcontinent through the same Sub-Saharan corridor used by cholera to enter Africa at the beginning of the 7^th ^pandemic [36]. During the '70s it spread from the Horn of Africa to Senegal, Guinea Bissau and eventually arrived in Angola: the new atypical variant might have disseminated by a similar route. This supposition might find some confirmation in the analysis performed by Sharma and colleagues who proposed the spread of a distinct *V. cholerae *O1 strain from India to Guinea Bissau, where it was associated with an epidemic of cholera in 1994 [22]. This hypothesis was based on the ribotype analysis of pre- and post- O139 *V. cholerae *O1 strains circulating in both countries. Our ribotype analysis confirmed these data since the Angolan strain from 2006, the clinical strains isolated in Guinea Bissau in 1994/1995 [37], and clinical post-O139 *V. cholerae *O1 strains from India [22] share the same profile, suggesting a common clonal origin. Unfortunately, the genetic content of the strains isolated in Guinea Bissau, in terms of ICE structure and CTXΦ array, was never investigated and our speculations cannot go any further.

Whichever route of dissemination used by the new variant to spread from the Indian Subcontinent to Africa, many evidences indicate that atypical *V. cholerae *strains are in the process of globally replacing the prototype El Tor strains, as observed in Angola.

## Conclusions

Cholera remains a global threat to public health and the recent outbreak in Haiti is a distressing example of this situation [38]. In 2006, Angola, which had reported no cholera cases since 1998, was affected by a major outbreak due to an atypical *V. cholerae *O1 El Tor strain that was analyzed for the first time in our study. This altered El Tor strain holds an RS1-CTX array on the large chromosome and a Classical *ctxB *allele and likely replaced the previous prototype O1 El Tor strain reported till 1994. The success of the new variant might depend on the combination of the respective predominant features of the El Tor and Classical biotypes: a better survival in the environment [2] and the expression of a more virulent toxin [39].

## Authors' contributions

The project was conceived and designed by DC, PC and MMC. All experiments were performed by DC and MS with the help of DB (ribotyping). The paper was written by DC, MS, PC, and MMC. All authors discussed the results, read and approved the final manuscript.

## Supplementary Material

Additional file 1 **Table S1. Amplicon profiles obtained for CTXΦ array A and B**. We designed new primer pairs able to discriminate between the different CTXΦ array on the chromosome of *V. cholerae*. In this table we present the region amplified by each primer pair and the two different arrays obtained for the strains under analysis. Click here for file
