# Supplementary material for: New V. cholerae atypical El Tor variant emerged during the 2006 epidemic outbreak in Angola
Source: BMC Microbiol. 2011 Jun 13;11:130. doi: 10.1186/1471-2180-11-130 (PMC3131240; doi:10.1186/1471-2180-11-130)
Supplement: Additional file 1 — Table S1. Amplicon profiles obtained for CTXΦ array A and B. We designed new primer pairs able to discriminate between the different CTXΦ array on the chromosome of V. cholerae. In this table we present the region amplified by each primer pair and the two different arrays obtained for the strains under analysis. [file 1471-2180-11-130-S1.DOC]

| **Primer pair** | **Region** | **CTX profile A** | **CTX profile B** |
| --- | --- | --- | --- |
| tlcF/rstAR | TLC-RS1/RS2 | 1740bp | 1740bp |
| tlcF/rstCR | TLC-RS1 | 3000bp | - |
| rstCF/rstAR | RS1-RS1/RS2 | 1095bp | - |
| ctxAF/rstAR | CORE-RS1/RS2 | - | 2087bp |
| rstCF/rtxR | RS1-RTX | - | 570bp |
| ctxAF/rtxR | CORE-RTX | 1542bp | 4267bp |
| ctxAF/cepR | CTX tandem array detection | - | - |
| chr2F/chr2R | Chromosome 2 CTX empty site | 1943bp | 1943bp |

**Table S1. Amplicon profiles obtained for CTXΦ array A and B.**
